# The Surgical Treatment of Dilated Stomach

**Published:** 1902-12-13

**Authors:** Leonard A. Bidwell

**Affiliations:** Surgeon to the Hospital, Dean of the Postgraduate College


### Hospital Clinics and Medical Progress.

### THE SURGICAL TREATMENT OF DILATED STOMACH.

A Postgraduate Lecture delivered at the West London Hospital. By Leonard A. Bidwell, F.R.C.S., Surgeon to the Hospital, Dean of the Postgraduate College.

Before actually considering the treatment I wish to briefly discuss the causes of dilatation, and some points in its diagnosis. The following are the principal causes of dilatation of the stomach :—(1) Cicatrical contraction of the pylorus, due usually to old gastric ulcer or to the swallowing of corrosive liquids; (2) malignant disease of the pylorus; (3) adhesions outside the pylorus, usually due to gall-stones; (4) spasm of the pylorus from ulcer or from excess of free hydrochloric acid; (5) gastroptosis; (6) ? atony; (7) various neuroses, and (8) floating kidney on the right side. I shall only refer to the first four named in this paper. Before passing on to the methods of diagnosis I should like to call your attention to the shape and position of the stomach. In the first place the stomach is not placed transversely in the abdomen, but the direction of its axis is nearly vertical, and in the normal stomach, when not distended, the pylorus is the lowest point of the stomach. The cardiac orifice is situated just to the left of the middle line, and the pyloric orifice is about half an inch to the right of the middle line, and is about three and a half inches below the cardia, the lesser curvature being nearly vertical. It is thus apparent why any corrosive acid affects the neighbourhood of the pylorus when swallowed, instead of the greater curvature. Secondly, as to the part of the stomach which is affected in dilatation. The dilatation does not affect the whole stomach, but is confined to that part of the organ known as the pyloric antrum, namely, the two inches just above the pylorus. Dilatation cannot take place upwards, and so must do so in the downward direction, with the result that a regular pouch is formed at a very considerably lower level than the pylorus, which is not permitted much movement, owing to the fixation of the duodenum. It therefore follows that, as the pylorus is no longer the lowest point of the stomach, it will be impossible for the organ to empty itself, and stasis of food will result. The result of this will be decomposition and excess of hydrochloric acid, which in its turn will produce spasm of the pylorus, and this will cause further dilatation. In this way an organic dilatation of the stomach can exist without any contraction of the pylorus. Little need be said here with regard to the methods of examination of a dilated stomach; inspection is of importance, since it is not uncommon to see waves of peristalsis passing over the dilated organ, and the peristaltic waves can be distinguished from those seen in intestinal obstruction, since in the former case the wave passes from left to right, and in the latter from right to left. Nothing need be said about palpation and percussion, since they are not of much importance unless a tumour be present. With regard to auscultation, besides hearing splashing and bubbling sounds over the pyloric region, it is affirmed that the stomach can be marked out by placing the stethoscope over the stomach area and then gently scratching the abdominal parietes with the finger; the scratching will be heard distinctly over the stomach area, and faintly when this part is left. I have tried this in a number of cases, but I do not think that it gives any reliable information. One of the most important methods of examination, however, is that of chemical analysis of the stomach contents. In every case of dilatated stomach a stomach tube should be used. It is preferable before passing it to give the patient a test breakfast of a roll and a pint of water or weak tea; half an hour afterwards a soft rubber tube should be swallowed by the patient; after withdrawing the stomach contents and setting them aside for future examination a Higginson's syringe should be attached to the stomach tube and air pumped in until the stomach is fully distended and its outline can be readily defined by palpation and percussion. The air must be let out before withdrawing the tube. The method is much preferable to that of distending the stomach by the generation of carbonic acid gas therein, which is effected by giving the two portions of a small seidlitz powder separately. This causes discomfort, since there is no means of regulating the amount of gas generated, and, moreover, carbonic acid gas causes spasm of the pylorus, and so entails additional discomfort.

The next step is the chemical analysis of the stomach contents. What we want to discover is whether free hydrochloric acid is present in excess or whether it is absent; and, secondly, whether lactic acid or acid lactates are present. The simplest test for free hydrochloric acid is a solution of phloroglaucin and vanillin in absolute alcohol: a few drops of this are placed on a china plate, a drop of the stomach filtrate is placed close to this, so that the two drops coalesce; the plate is then warmed, and if free hydrochloric acid is present a red tinge appears. The easiest test for acid lactates or lactic acid is to place a few drops of neutral ferric chloride solution in 3 drachms of 1 in 20 carbolic acid solution and then add water till an amethyst blue colour results; a few drops of the stomach filtrate added to this produces a yellow colorisation if lactic acid is present. The importance of these tests lies in the fact that excesss of hydrochloric acid occurs during gastric ulcer or in dilatation from cicatricial contraction; while in cases of malignant disease free hydrochloric acid is absent, but lactic acid or acid lactates are present. Unfortunately, however, these differences are not constant, and the diagnosis cannot be positive on these grounds.

I will now pass on to the signs which would make us suspect that dilatation of the stomach was due to malignant disease. Briefly, the symptoms of malignant disease of the pylorus are: (1) Pain usually localised to the pyloric region and also felt in the back; (2) signs of dilatation of the stomach; (3) retching and vomiting usually only after solids; (4) peristaltic waves seen over the stomach area; (5) absence of free hydrochloric acid from the fluid obtained on passage of the tube, or from the vomited matter; (6) progressive emancipation; (7) obstinate constipation; and (8) progressive anæmia; to these may be added the presence of a tumour and coffee-ground vomiting. These last two symptoms, however, often are not seen till late in the case, and did we wait for their appearance, we should let slip by the time when surgical interference could do good. The symptoms in dilatation due to non-malignant structure are rather different. Then the pain is not so severe; (2) the dilatation is often more extreme; (3) there is not so much retching and the vomiting at first is at more or less distant intervals, and this is very large in amount, followed by complete relief for some days at first; (4) peristaltic waves are not so often found; (5) free hydrochloric acid is present in the stomach contents and is often in excess; (6) the emanciation is not so rapid; (7) the constipation even more marked; and (8) anæmia is absent or little marked. Coffee-ground vomiting is seldom present, and a tumour is rarely felt.

(*To be Continued.*)

